# Supplementary material for: Roles of Tubulin Concentration during Prometaphase and Ran-GTP during Anaphase of C. elegans meiosis
Source: bioRxiv. 2024 Jun 25:2024.04.19.590357. Originally published 2024 Apr 20. Preprint. [Version 2] doi: 10.1101/2024.04.19.590357 (PMC11042349; doi:10.1101/2024.04.19.590357)
Supplement: Supplement 1 [file media-1.pdf]

Supplemental Data 1: Sequences of ran degrons:

ran-3(syb7781) II:
GGAAAAGTGTTTGCTATGGGAAAGAACACAGACAATGCTCTCGGCCTCGGTAATTG
GACTGGAAAGGACGACCAACAGCATTGGTTGTACGATACAATCCAGGAAATAGAATT
CGATTCGAAGATCGTTGGTGTTTCTGCCAAACTAGCCACTTCTATCGCCTGGTCTGA
GGATGGAACCGCCTACGCTTGGGGTTTTGATACTACCGGACAACTTGGTCTCGGAT
TGAAAGACGAAGACGAGAAGgtaatttttcaaaactcaaactatcaataaataaaatattcaatcttattttccag
ATGGTGTCCAAGCCAGAGGAGATCAGCTCC**GCACAC**CTTGACGGTTATAGTATTAT
CGGGGCTTCGATTTCCGATCAGCACACTTTGATTATTGCCAAGAAAAATGGAGCATC
GGGAGCCTCAGGAGCATCGATGCCTAAAGATCCAGCCAAACCTCCGGCCAAGGCA
CAAGTTGTGGGATGGCCACCGGTGAGATCATACCGGAAGAACGTGATGGTTTCCTG
CCAAAAATCAAGCGGTGGCCCGGAGGCGGCGGCGTTCGTGAAGGGAGCATCGGG
AGCCTCAGGAGCATCGATGGCTGAAATTGGCACAGGATTCCCGTTTGACCCCCACT
ACGTCGAGGTCCTCGGAGAGCGTATGCACTACGTCGACGTCGGACCACGTGACGG
AACCCCAGTCCTCTTCCTCCACGGAAACCCAACCTCCTCCTACGTCTGGCGTAACA
TCATCCCACACGTCGCCCCAACCCACCGTTGCATCGCCCCAGACCTCATCGGAATG
GGAAAGTCCGACAAGCCAGACCTCGGATACTTCTTCGACGACCACGTCCGTTTCAT
GGACGCCTTCATCGAGGCCCTCGGACTCGAGGAGGTCGTCCTCGTCATCCACGAC
TGGGGATCCGCCCTCGGATTCCACTGGGCCAAGCGTAACCCAGAGCGTGTCAAGgt
aagtttaaacatatatatactaactaaccctgattatttaaattttcagGGAATCGCCTTCATGGAGTTCATCC
GTCCAATCCCAACCTGGGACGAGTGGCCAGAGTTCGCCCGTGAGACCTTCCAAGC
CTTCCGTACCACCGACGTCGGACGTAAGCTCATCATCGACCAAAACGTCTTCATCG
AGGGAACCCTCCCAATGGGAGTCGTCCGTCCACTCACCGAGGTCGAGATGGACCA
CTACCGTGAGCCATTCCTCAACCCAGTCGACCGTGAGCCACTCTGGCGTTTCCCAA
ACGAGCTCCCAATCGCCGGAGAGCCAGCCAACATCGTCGCCCTCGTCGAGGAGTA
CATGGACTGGCTCCACCAATCCCCAGTCCCAAAGCTCCTCTTCTGGGGAACCCCA
GGAGTCCTCATCCCACCAGCCGAGGCCGCCCGTCTCGCCAAGTCCCTCCCAAACT
GCAAGgtaagtttaaacagttcggtactaactaaccatacatatttaaattttcagGCCGTCGACATCGGACC
AGGACTCAACCTCCTCCAAGAGGACAACCCAGACCTCATCGGATCCGAGATCGCC
CGTTGGCTCTCCACCCTCGAGATCTCCGGATAAattatttggttttattctcaactttatatcagttttgtttt
gtctctgtagcattattttgttattttttcgtttcccggtagccaattcgattgttctccagtaacattctcatcaatttttctgttttttttatc
atttcatttttgtcaagtagcatcagtcatgtaagaaagggatagagttcccttctgtgaaaatggagaattgttgaaacgtcgt
tgcacaacgacgtttaaacacttcactctatccctggattgatttccaacttctatgttttctcaaaacccccctattagttctgctat
atattggaatccaaaaatttttcatttttagcctgagttatgttgttctcttatcatgtgaactcactgttttataatccgtttcaccattt
atggtaaacgtttcctgg

3’ of ran-3 are highlighted in yellow (within which synonymous mutation is labeled in blue text); AID in red text followed by halo sequence in blue text. Linker sequences (in purple) are inserted among the genes.

ran-2(syb7819) III:
AGCTATTGAAGTTGCAGgtaagaaatataaaaatattttaatataactactttcaatttttaagAAAATATCGT
CCGCCGAGTGGAGTCTGTCAAGCGTAACCCGATTCCGGCCACAACTCAATTAGTTA
ACAATATTGTTGCTCAATGTGCAGGAACAGGAGTTAAGgtaggtatttttcaagcttattctaaaaaa
cgtttaatatatgagacagactttacagGCTGAAACTGATTGGGGATATGGTGCCGATCCACAAG
TGATTTCACGTTTGTTCTCGGAACTTGTTGCTCGCGGCCATTTCAAG**C****T****T**GAGCTG
GCTCTCCTTCAACGCTTTTTCgtaagtctcacaactatattttatggttattttttcaatttttcagCCTTCACA
AGGAGCATCGGGAGCCTCAGGAGCATCGATGCCTAAAGATCCAGCCAAACCTCCG
GCCAAGGCACAAGTTGTGGGATGGCCACCGGTGAGATCATACCGGAAGAACGTGA
TGGTTTCCTGCCAAAAATCAAGCGGTGGCCCGGAGGCGGCGGCGTTCGTGAAGG
GAGCATCGGGAGCCTCAGGAGCATCGATGGCTGAAATTGGCACAGGATTCCCGTT
TGACCCCCACTACGTCGAGGTCCTCGGAGAGCGTATGCACTACGTCGACGTCGGA
CCACGTGACGGAACCCCAGTCCTCTTCCTCCACGGAAACCCAACCTCCTCCTACG
TCTGGCGTAACATCATCCCACACGTCGCCCCAACCCACCGTTGCATCGCCCCAGAC
CTCATCGGAATGGGAAAGTCCGACAAGCCAGACCTCGGATACTTCTTCGACGACCA
CGTCCGTTTCATGGACGCCTTCATCGAGGCCCTCGGACTCGAGGAGGTCGTCCTC
GTCATCCACGACTGGGGATCCGCCCTCGGATTCCACTGGGCCAAGCGTAACCCAG
AGCGTGTCAAGgtaagtttaaacatatatatactaactaaccctgattatttaaattttcagGGAATCGCCTTC
ATGGAGTTCATCCGTCCAATCCCAACCTGGGACGAGTGGCCAGAGTTCGCCCGTG
AGACCTTCCAAGCCTTCCGTACCACCGACGTCGGACGTAAGCTCATCATCGACCAA
AACGTCTTCATCGAGGGAACCCTCCCAATGGGAGTCGTCCGTCCACTCACCGAGG
TCGAGATGGACCACTACCGTGAGCCATTCCTCAACCCAGTCGACCGTGAGCCACT
CTGGCGTTTCCCAAACGAGCTCCCAATCGCCGGAGAGCCAGCCAACATCGTCGCC
CTCGTCGAGGAGTACATGGACTGGCTCCACCAATCCCCAGTCCCAAAGCTCCTCTT
CTGGGGAACCCCAGGAGTCCTCATCCCACCAGCCGAGGCCGCCCGTCTCGCCAA
GTCCCTCCCAAACTGCAAGgtaagtttaaacagttcggtactaactaaccatacatatttaaattttcagGCC
GTCGACATCGGACCAGGACTCAACCTCCTCCAAGAGGACAACCCAGACCTCATCG
GATCCGAGATCGCCCGTTGGCTCTCCACCCTCGAGATCTCCGGATAAtcgagcttctctac
acgatatcccagtcttcgcgatttttttacatgatttcagataagccgtggttattttatatttggatctacaatacatgtattattcat
cgtatcgtgaacaatatatttcttcaacttccaatgtatacacgagttatcaatttgttttaattttgtttctctaatttccactaattttta
gtttaatactttaaatctctttcttgtacgtgtaaatgcttcaatccgtttttcaagtaaatttttgtgaacgaagttgtttttatgattac
atgttttatgtctttgtgaacttttttt

3’ of ran-2 are highlighted in yellow (within which synonymous mutation is labeled in blue text); AID in red text followed by halo sequence in blue text. Linker sequences (in purple) are inserted among the genes.

Supplemental Data 2: Sequences of transgene insertions:

1. Sequence of GFP::GCN4-pLi:

ATGCCT**GCATcc**TCCAAGGGAGAGGAGCTCTTCACCGGAGTCGTCCCAATCCTCGTCGAGCTCGACGGAGgtattttcctgcatttttcaact
gggaaaatgaaagaaaatcgataatttcagACGTCAACGGACACAAGTTCTCCGTCTCCGGAGAGGGAGAGGGAGACGCCACCTACGGAAA
GCTCACCCTCAAGTTCATCTGCACCACCGGAAAGCTCCCAGTCCCATGGCCAACCCTCGTCACCACCTTCTGCTACGGAGgtaag
atatgggaaagaaggaaaaaaccgagattttacttgaaaaattgaatttttcgcgggattttcaccaaaaattgttgaatattcattatttcacgctgtaaaacaaaaaaaaaaaaaatcaaa
aactacgttgaaatcgcgtttttaagcgaattttcttcagaattgccagattttaaccccaaattttgcagtttttaaataaaatttcacctttttcggctcaaattgtagattttcctgaaaatttagtacaa
aaaacaatttcctcgtaaatttttcaaattttcagTCCAATGCTTCTCCCGTTACCCAGACCACATGAAGCGTCACGACTTCTTCAAGTCCGCCATG
CCAGAGGGATACGTCCAAGAGCGTACCATCTTCTTCAAGGACGACGGAAACTACAAGACCCGTGCCGAGGTCAAGTTCGAGGG
AGACACCCTCGTCAACCGTATCGAGCTCAAGGGAATCGACTTCAAGGAGGACGGAAACATCCTCGGACACAAGCTCGAGTACA
ACTACAACTCCCACAACGTCTACATCATGGCCGACAAGCAAAAGAACGGAATCAAGGTCAACTTCAAGgtacggattgaaattgctttaaaatt
tgaaaaattgattaaaaagtgcatttttaagctttgaccgacttaaaattagatttctgagcctattttctgagaattggaattttttcattgtgaaagttcaaagaatagcgtgaatgattagaaaa
tattgtaaaatttcaatttttcctataaaaaaggatttttttaggaatcaaaaattgcaaaatgatgccctaaaattcgaaaataaatataaaaattggcggtttctcaaaaatctagaattccgac
cttaattattaatttttttacaatatttttttttgaaaaaaatccagaaaatttgaatttcgtagtttgtagtctaggcctctatcaattaaattttcgattttttgagtaaaatttcgaatttactattatttggacc
aaaattgtatttttttttcagaattaaatttataaaattttcagaaattaaatttataaaaaaaaaaaaaaaattaaaaaaaaaaaaaaaaaaattaaaaattaaaaattaaaaaattaagttttttta
ctcaaaattttgcactgaaattcgaaaatctaaaaatccgacctaaagtctgtatttttcaacaaaaattcagaaaaaactcaaaaacttgtattttgtagccagtcaaccactttctaaaatatc
aaatttgaatttttcaagcatttttacattgaaaaatctaatttttcgagtgaaaccacttgaaaatctgaaaattgaattaatttctgaactttagagatttttttgtcaaaattttagataaagggattttt
aaacaaaaaattgatttttaatcgaaaaatactgggatttatgggttttttaaaagaaaaacgggggtttgaatgaaaaatcgcctgaaatcttagaaaattaattaaaaaactatgatttaatt
ccaaaaattaccgaaaatatcaaattttccatttttaaaccctaaattctttcagATCCGTCACAACATCGAGGACGGATCCGTCCAACTCGCCGACCACTAC
CAACAAAACACCCCAATCGGAGACGGACCAGTCCTCCTCCCAGACAACCACTACCTCTCCACCCAATCCGCCCTCTCCAAGgtag
attttttagaatttttgggtttttgaagtagaaaatcataaaaatctagggtttttatgaattgttttgaagaaaaattgcaaaaattccacaaaatggaagaaaaataactttggaagcgcatttttc
gcaaaaaaaccgaaatttttgcgtaaaatttcaaaattgcaataaaaattccacaaacatcaaatttcttaaatttttttataaaaaattggatggaaacactcttgaatttagaaaaaaaatcag
tttttctcatctaaaacttcaaaatttcggtgtaatcccattaaaattgccacaaaattcggaaatttcacctgaaatagagtgaataatttaaaatgttcagaaattcatattttgtcattttaaaagc
attaaaacaaatcaaaaatctattttttttggttggaaaagttcaaaattctggagaaattacattaaaattttcgtaaaatatggtaaataaacgaaaatgttgagaaattaaagaaaagttaca
atttttagcttaaaaattcaacattttgaggaaatgccacctaaaaaagtgactaatcgaaaatgttgagaaattaaaattgccaccatttatttatataaactactctaaaattacaattttcatgtt
aaaaattaataaaaaatctactttttccaaactacagtaaccttaccgtatacctacagtacctgaacattgccccccaccagctcccaacccaatacctcctcaaaaacttacacctcaatttt
tcataaactacagtaaccctaccaaaaaagcacaaaaaaaaaatctacattcattttccaacaattttcaatattttcagGACCCAAACGAGAAGCGTGACCACATGGTC
CTCCTCGAGTTCGTCACCGCCGCCGGAATCACCCACGGAATGGACGAGCTCTACAAGCCTGCATTCaGGAGGcTCCGGtGGgTCg
GGtGGcTCaGGgGGAATGAAACAGATAGAGGATAAGCTTGAGGAGATTCTGTCCAAGCTGTATCACATTGAGAATGAGCTCGCTC
GCATCAAGAAGCTCTTGGGGGAGCGGTAA

GFP optimized for germline expression is highlighted in green. GCN4-pLi is colored red.

2. Sequence of GFP::GCN4-pLi with negative charge:

"GAAGATGAAGATGAGGACGAGGCA" was inserted onto GFP::GCN4-pLi before GCATcc (highlighted in bold)

3. Sequence of GFP::GCN4-pLi with neutral charge:

"CGCAGACGTAGACGTCGTCGAGCAGCT" was inserted onto GFP::GCN4-pLi before GCATcc (highlighted in bold)

4. Sequence of GFP::GCN4-pLi with positive charge:

**"CGCAGACGTAGACGTCGTCGAGCACGTCGTCGAGCACGTCGACGTGCCCGTGCTCGTCGTCGTCGAGCACGTCGTCGAGC
A"** was inserted onto GFP::GCN4-pLi before GCATcc (highlighted in bold)

5. Sequence of GFP::tba-2(T349E):
ATGCCTGCATccTCCAAGGGAGAGGAGCTCTTCACCGGAGTCGTCCCAATCCTCGTCGAGCTCGACGGAGgtattttcctgcatttttcaactg
ggaaaatgaaagaaaatcgataatttcagACGTCAACGGACACAAGTTCTCCGTCTCCGGAGAGGGAGAGGGAGACGCCACCTACGGAAAG
CTCACCCTCAAGTTCATCTGCACCACCGGAAAGCTCCCAGTCCCATGGCCAACCCTCGTCACCACCTTCTGCTACGGAGgtaagat
atgggaaagaaggaaaaaaccgagattttacttgaaaaattgaatttttcgcgggattttcaccaaaaattgttgaatattcattatttcacgctgtaaaacaaaaaaaaaaaaaatcaaaaa
ctacgttgaaatcgcgtttttaagcgaattttcttcagaattgccagattttaaccccaaattttgcagtttttaaataaaatttcacctttttcggctcaaattgtagattttcctgaaaatttagtacaaaa
aacaatttcctcgtaaatttttcaaattttcagTCCAATGCTTCTCCCGTTACCCAGACCACATGAAGCGTCACGACTTCTTCAAGTCCGCCATGCC
AGAGGGATACGTCCAAGAGCGTACCATCTTCTTCAAGGACGACGGAAACTACAAGACCCGTGCCGAGGTCAAGTTCGAGGGAG
ACACCCTCGTCAACCGTATCGAGCTCAAGGGAATCGACTTCAAGGAGGACGGAAACATCCTCGGACACAAGCTCGAGTACAACT
ACAACTCCCACAACGTCTACATCATGGCCGACAAGCAAAAGAACGGAATCAAGGTCAACTTCAAGgtacggattgaaattgctttaaaatttgaa
aaattgattaaaaagtgcatttttaagctttgaccgacttaaaattagatttctgagcctattttctgagaattggaattttttcattgtgaaagttcaaagaatagcgtgaatgattagaaaatattg
taaaatttcaatttttcctataaaaaaggatttttttaggaatcaaaaattgcaaaatgatgccctaaaattcgaaaataaatataaaaattggcggtttctcaaaaatctagaattccgacctta
attattaatttttttacaatatttttttttgaaaaaaatccagaaaatttgaatttcgtagtttgtagtctaggcctctatcaattaaattttcgattttttgagtaaaatttcgaatttactattatttggaccaaa

attgtatttttttttcagaattaaatttataaaattttcagaaattaaatttataaaaaaaaaaaaaaaattaaaaaaaaaaaaaaaaaattaaaaattaaaaattaaaaaattaagtttttttttactca
aaattttgcactgaaattcgaaaatctaaaaatccgacctaaagtctgtatttttttcaacaaaaattcagaaaaaactcaaaaacttgtattttgtagccagtcaaccactttctaaaatatcaaat
ttgaatttttcaagcatttttacattgaaaaatctaatttttcgagtgaaaccacttgaaaatctgaaaattgaattaatttctgaactttagagatttttttgtcaaaattttagataaagggatttttaaac
aaaaaattgatttttttaatcgaaaaatactgggatttatgggtttttttaaaagaaaaacgggggtttgaatgaaaaatcgcctgaaatcttagaaaattaattaaaaaactatgatttaattccaa
aaattaccgaaaatatcaaattttccatttttaaaccctaaattctttcagATCCGTCACAACATCGAGGACGGATCCGTCCAACTCGCCGACCACTACCAA
CAAAACACCCCAATCGGAGACGGACCAGTCCTCCTCCCAGACAACCACTACCTCTCCACCCAATCCGCCCTCTCCAAGgtagattttttt
agaatttttgggtttttgaagtagaaaatcataaaaatctagggtttttatgaattgttttgaagaaaaattgcaaaaattccacaaaatggaagaaaaataactttggaagcgcatttttcgcaa
aaaaaccgaaatttttgcgtaaaatttcaaaattgcaataaaaattccacaaacatcaaatttcttaaattttttataaaaaattggatggaaacactcttgaatttagaaaaaaaatcagtttttct
catctaaaacttcaaaatttcggtgtaatcccattaaaattgccacaaaattcggaaatttcacctgaaatagagtgaataatttaaaatgttcagaaattcatattttgtcattttaaaagcattaa
aacaaatcaaaaatctattttttttggttggaaaagttcaaaattctggagaaattacattaaaattttcgtaaaatatggtaaataaacgaaaatgttgagaaattaaagaaaagttacaattttt
agcttaaaaattcaacattttgaggaaatgccacctaaaaaagtgactaatcgaaaatgttgagaaattaaaattgccaccatttatttatataaactactctaaaattacaattttcatgttaaaa
attaataaaaaatctactttttccaaactacagtaaccttaccgtatacctacagtacctgaacattgccccccaccagctcccaacccaatacctcctcaaaaacttacacctcaatttttcata
aactacagtaaccctaccaaaaaagcacaaaaaaaaaatctacattcattttccaacaattttcaatattttcagGACCCAAACGAGAAGCGTGACCACATGGTCCTC
CTCGAGTTCGTCACCGCCGCCGGAATCACCCACGGAATGGACGAGCTCTACAAGCCTGCAATGCAATGCGTGAGGTCATCTCTA
TCCACGTCGGACAAGCCGGAGTCCAAATCGGAAACGCCTGCTGGGAGCTCTACTGCCTCGAGCACGGAATCCAGCCCGATGG
AACCATGCCAACTCAATCAACGAACGAGGGAGAGTCGTTCACCACTTTCTTCTCAGACACCGGATCCGGCCGTTACGTTCCAAG
ATCCATCTTCGTCGATCTCGAGCCAACTGTCGTTGACGAGATTCGCACTGGAACCTACAAGAAGCTCTTCCATCCAGAGCAGATG
ATCACCGGAAAGGAAGACGCCGCTAACAACTACGCTCGTGGACACTACACCGTCGGAAAGGAGCTCATCGACACCGTCCTCGA
CAGGATCCGTCGTCTCGCTGATAACTGCAGTGGACTCCAAGGATTCTTCGTCTTCCACTCCTTCGGAGGAGGTACCGGATCCGG
ATTCACTTCGCTTCTTATGGAACGTCTTTCCGTCGACTACGGAAAGAAGTCCAAGCTCGAGTTCTCCATCTACCCAGCTCCACAG
GTCTCAACCGCCGTCGTTGAGCCATACAACTCGATCCTCACCACCCATACCACCTTGGAGCACTCCGACTGCGCCTTCATGGTC
GATAACGAGGCCATCTACGACATCTGCCGCAGAAACTTGGATGTTGAGCGACCAAGCTACACCAACCTCAACAGAATCATCTCCC
AGgtttgttgagctcaatttgattgttgtattctaatgttctctttttacagGTTGTCTCCTCAATCACTGCTTCCTTGAGATTCGATGGAGCCCTCAACGTTGAT
CTCAACGAGTTCCAGACCAACTTGGTGCCATACCCAAGAATTCACTTCCCATTGGCCGCCTACACTCCACTCATCTCTGCTGAGA
AGGCCTACCACGAGGCTCTGTCCGTCAGCGACATCACCAATAGCTGCTTCGAGCCGGCTAACCAGATGGTCAAGTGTGATCCAC
GTCACGGAAAGTACATGGCTGTGTGCCTCTTGTACAGAGGAGACGTCGTTCCAAAGGACGTTAACACCGCCATCGCTGCAATCA
AGACCAAGAGAACCATCCAATTCGTCGATTGGTGCCCA**GAG**GGATTCAAGGTCGGAATCAACTACCAGCCACCAACTGTTGTGC
CAGGAGGTGATCTTGCCAAGGTGCCACGCGCCGTCTGCATGCTCTCCAACACTACCGCCATCGCTGAGGCCTGGTCTCGTCTC
GACTACAAGTTCGACTTGATGTACGCCAAGCGTGCCTTCGTCCACTGgtatgttgcccgttaaactatacttttcaaatattcaatgtgtttcttttcagGTACG
TCGGAGAAGGTATGGAGGAAGGAGAGTTCACCGAGGCTCGTGAGGACTTGGCTGCTCTCGAGAAGGACTACGAAGAGGTCGG
AGCTGACTCCAACGAGGGAGGAGAAGAGGAGGGAGAGGAGTACTAGCGCGTCGCGTAAaTAAaTAA

GFP optimized for germline expression is highlighted in green. tba-2(T349E) is colored red. T349E mutation is emphasized in bold text.

Table S1: *C. elegans* strains used in this study.

Strain name	Genotype	Source
FM917	*fxIs1 [pie-1p::TIR1::mRuby, I:2851009] I;*<br>*ltIs37 [pAA64; pie-1p::mCh::his-58 + unc-119(+)];*<br>*ruIs57 [pie-1p::GFP::tubulin + unc-119(+)] V*	This study
FM1054	*fxIs1[pie-1p::TIR1::mRuby, I:2851009] I;*<br>*ran-3(syb7781[ran-3-3xGAS-AID-3xGAS-HALO]) II;*<br>*ltIs37 [pAA64; pie-1p::mCh::his-58 + unc-119(+)];*<br>*ruIs57 [pie-1p::GFP::tubulin + unc-119(+)] V*	This study
FM1056	*fxIs1[pie-1p::TIR1::mRuby, I:2851009] I;*<br>*ran-2(syb7819[ran-2-3xGAS-AID-3xGAS-HALO]) III;*<br>*ltIs37 [pAA64; pie-1p::mCh::his-58 + unc-119(+)];*<br>*ruIs57 [pie-1p::GFP::tubulin + unc-119(+)] V*	This study
FM717	*bus-17(e2800)X;*<br>*ltSi1412 [pNA20; Pmex-5::mNeonGreen::tbb-2 operon linker mCh::his-11::Ptbb-2; cb-unc-199(+)]I;*<br>*unc-119(ed3)III clone B (MOS I insertion)*	This study
NM5402	*jsSi1579 [loxP::rpl-28p::FRT::GFP::his-58 FRT3] II.*<br>*bqSi711 [mex-5p::FLP::SL2::mNG + unc-119(+)] IV.*	Gifted from Nonet Lab
FM971	*duSi18[GFP(SMU)-GCN4-pLI] II;*<br>*ltIs37 [pAA64; pie-1p::mCh::his-58 + unc-119(+)];*<br>*him-8(e1489)*	This study
FM1011	*duSi20[GFP(SMU)::tba-2(T349E)] II*<br>*ltIs37 [pAA64; pie-1p::mCh::his-58 + unc-119(+)];*<br>*him-8(e1489)*	This study
FM628	*unc-119(ed3) III;*<br>*ltSi464[pNH103; Pmex-5::npp6::GFP::tbb-2 3'UTR; cbunc-119(+)] I;*<br>*ltIs37[pAA64; pie-1::mCherry::his-58; unc-119 (+)] IV*	A gift from Oegema-Desai Lab
BN359	*ima-2(ok256) I/hT2[bli-4(e937) let-?(q782) qIs48] (I;III);*<br>*qaIs3502[pie-1p::YFP::lmn-1 + pie-1p::CFP::H2B + unc-119(+)]*	CGC
FM991	*wjIs76[Cn_unc-119(+); pie-1p::mKate2::tba-2];*<br>*vit-2(crg9070[vit-2::gfp]) X;*<br>*egxSi126 [mex-5p::hsp-3(aa1-19)::halotag::HDEL::pie-1 3'UTR+ unc-119(+)] I. "*	This study
FM691	*cox-4(zu476[cox-4::eGFP::3xFLAG]) I;*<br>*wjIs76[Cn_unc-119(+); pie-1p::mKate2::tba-2]*	This study
CZ18550	*juSi123[rpl-29::GFP] II; rpl-29(tm3555) IV*	CGC
ABR5	*staIs1 [pie-1p::GFP + unc-119(+)];*<br>*unc-119(ed3) III*	CGC

FM1103	*duSi21[HALO(smu)] II;*<br>*ruIs57 [pie-1p::GFP::tubulin + unc-119(+)] V*<br>*itIs37 [pie-1p::mCh::H2B::pie-1 3'UTR + unc-119(+)] IV"*	This study
FM1168	*duSi23[minus7-GFP(SMU)::GCn4-pLI] II;*<br>*ItIs37[pAA64; pie-1::mCherry::his-58; unc-119 (+)] IV*	Negative Charge; this study
FM1169	*duSi24[plus7-GFP(SMU)::GCn4-pLI] II;*<br>*ItIs37[pAA64; pie-1::mCherry::his-58; unc-119 (+)] IV*	Neutral Charge; this study
FM1180	*duSi25[plus21-GFP(SMU)::GCn4-pLI] II;*<br>*ItIs37[pAA64; pie-1::mCherry::his-58; unc-119 (+)] IV*	Positive charge; this study
