# Supplementary material for: Mechanisms of Meiotic Spindle Initiation in Caenorhabditis elegans Oocytes
Source: bioRxiv. 2024 Apr 20:2024.04.19.590357. Preprint. [Version 1] doi: 10.1101/2024.04.19.590357 (PMC11042349; doi:10.1101/2024.04.19.590357)
Supplement: Supplementary file 1 [file NIHPP2024.04.19.590357V1-supplement-1.pdf]

bioRxiv preprint doi: https://doi.org/10.1101/2024.04.19.590357; this version posted April 20, 2024. The copyright holder for this preprint (which was not certified by peer review) is the author/funder, who has granted bioRxiv a license to display the preprint in perpetuity. It is made available under aCC-BY 4.0 International license.

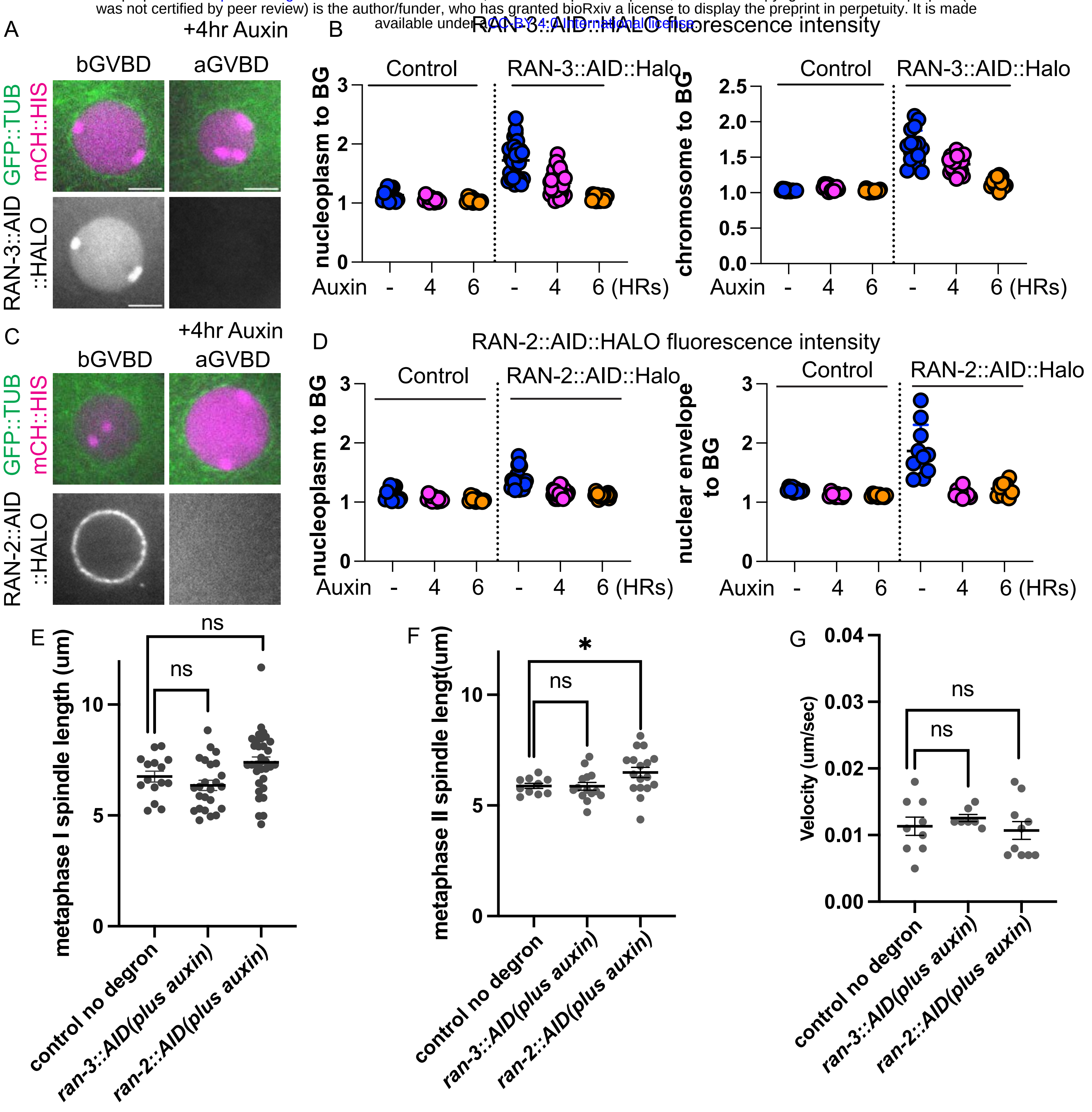

Fig S1. Ran-GEF and Ran-GAP are not required for meiotic spindle assembly. (A) Images of RAN-3::AID::HALO, TIR1::mRuby, GFP::TUB and mCh::histone -1 oocytes before Germinal Vesicle Breakdown (bGVBD) and after (aGVBD). (B) Fluorescence intensity ratios of HALO in oocytes treated with 0, 4 or 6-hour Auxin. Control: strain not containing AID::HALO tag. (C) Images of RAN-2::AID::HALO, TIR1::mRuby, GFP::TUB and mCh::histone -1 oocytes before Germinal Vesicle Breakdown (bGVBD) and after (aGVBD). (D) Fluorescence intensity ratios of HALO in oocytes treated with 0, 4 or 6-hour Auxin. Control: strain not expressing AID::HALO. (E) Metaphase I spindle lengths determined from time-lapse. (F) Metaphase II spindle lengths determined from time-lapse. (G) Anaphase II velocities determined from time-lapse. ns P > 0.05, *P ≤ 0.05, **P ≤ 0.01, ***P ≤ 0.001, ****P ≤ 0.0001, Mann-Whitney U test. Size Bars, 5um.

bioRxiv preprint doi: https://doi.org/10.1101/2024.04.19.590357; this version posted April 20, 2024. The copyright holder for this preprint (which was not certified by peer review) is the author/funder, who has granted bioRxiv a license to display the preprint in perpetuity. It is made available under aCC-BY 4.0 International license.

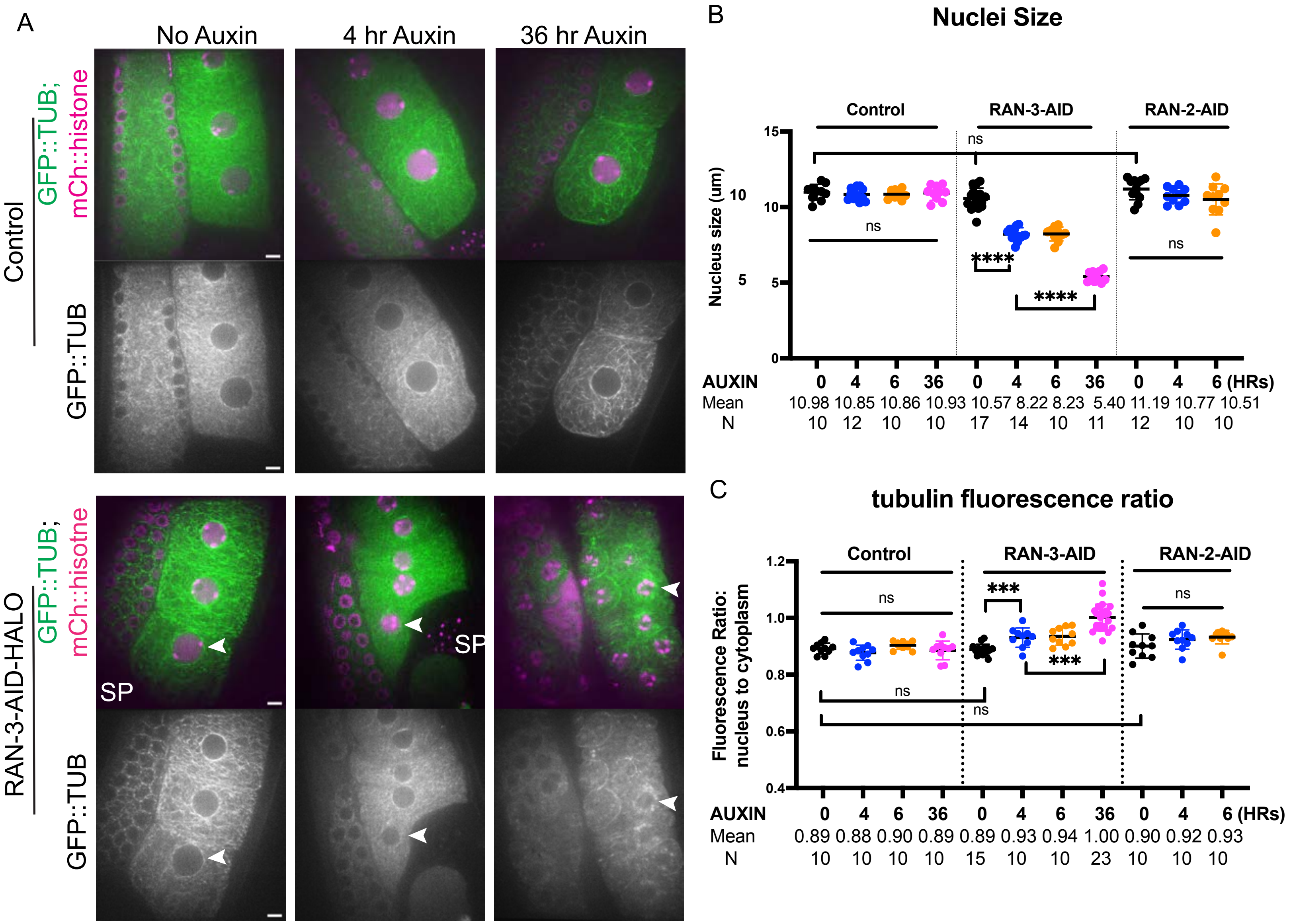

Fig S2. RAN-3::AID::HALO oocytes treated with auxin have pre-GVBD defects.
(A) Representative images of oocytes expressing TIR1::mRuby, GFP::TUB (tubulin) and mCh::histone (mCherry) or RAN-3::AID::HALO, TIR1::mRuby, GFP::TUB and mCh::histone, treated with no auxin, 4hr auxin or 36hr auxin, respectively. Scale bars, 5μm.
(B) Quantification of oocyte nuclei size.
(C) Quantification of microtubule fluorescence ratio in nucleus to cytoplasm as an indication of nuclear envelope leakiness. ns P > 0.05, *P ≤ 0.05, **P ≤ 0.01, ***P ≤ 0.001, ****P ≤ 0.0001, Mann-Whitney U test. Size Bars, 5um.

bioRxiv preprint doi: https://doi.org/10.1101/2024.04.19.590357; this version posted April 20, 2024. The copyright holder for this preprint (which was not certified by peer review) is the author/funder, who has granted bioRxiv a license to display the preprint in perpetuity. It is made available under aCC-BY 4.0 International license.

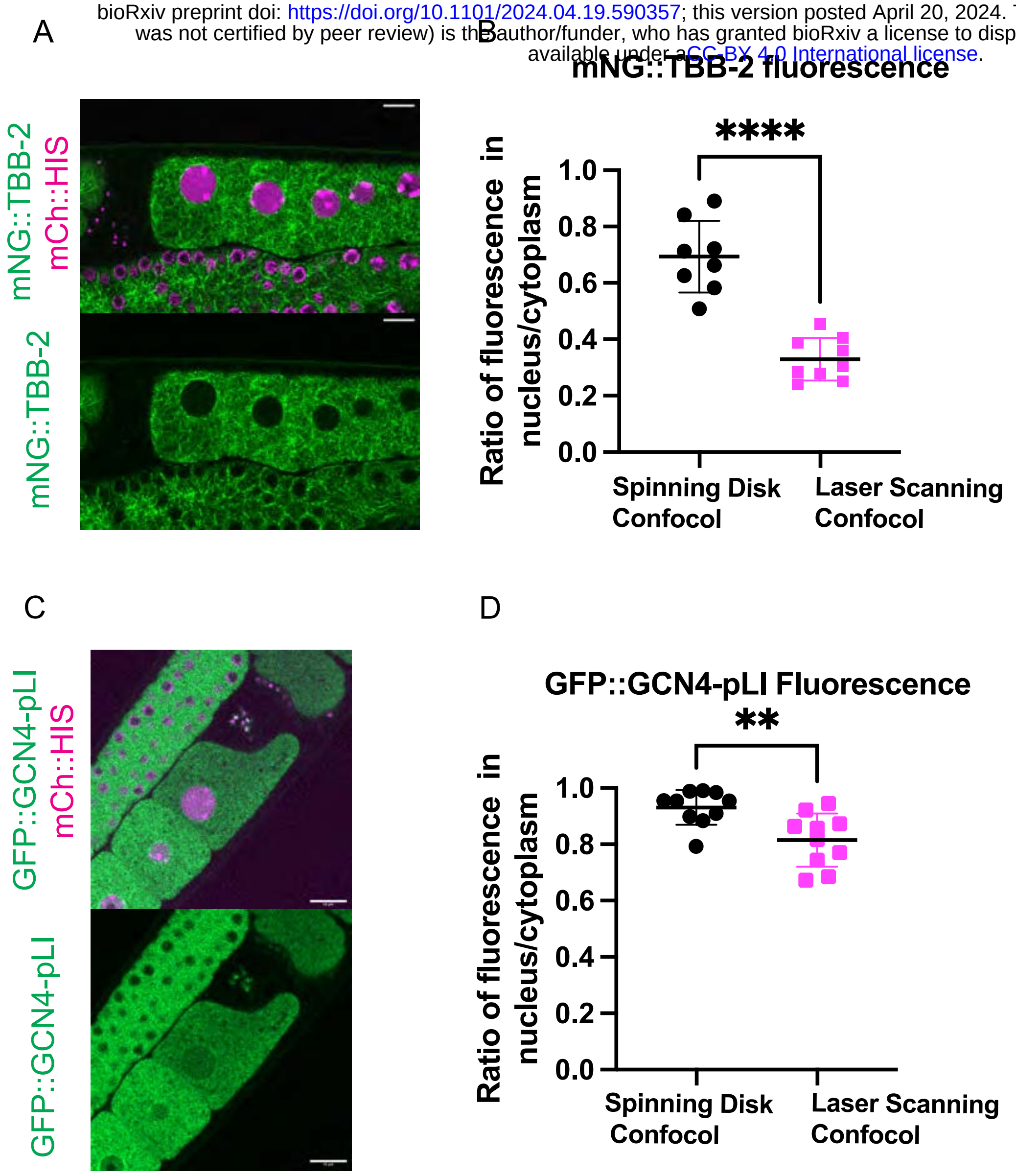

Fig S3. Difference of tubulin or GCN4-pLI fluorescence between nucleus and cytoplasm measured from Zeiss LSM confocal microscope is greater than from spinning disk confocal.

(A) Representative images of diakinesis oocytes expressing mNG::TBB-2 (mNeonGreen:: tubulin) and mCh::HIS (mCherry::histone H2b) from Zeiss LSM confocal microscope. Scale bars, 10μm.

(B) Comparison of fluorescence intensity ratios of nucleus/cytoplasm in images acquired with a spinning disk confocal vs a laser scanning confocal.

(C) Representative images of oocytes expressing GFP::GCN4-pLI and mCh::HIS from Zeiss LSM confocal microscope. Scale bars, 10μm.

(D) Ratio of GFP::GCN4-pLI fluorescence in the nucleus/cytoplasm from images captured on a spinning disk confocal vs a laser scanning confocal.

bioRxiv preprint doi: https://doi.org/10.1101/2024.04.19.590357; this version posted April 20, 2024. The copyright holder for this preprint (which was not certified by peer review) is the author/funder, who has granted bioRxiv a license to display the preprint in perpetuity. It is made available under aCC-BY 4.0 International license.

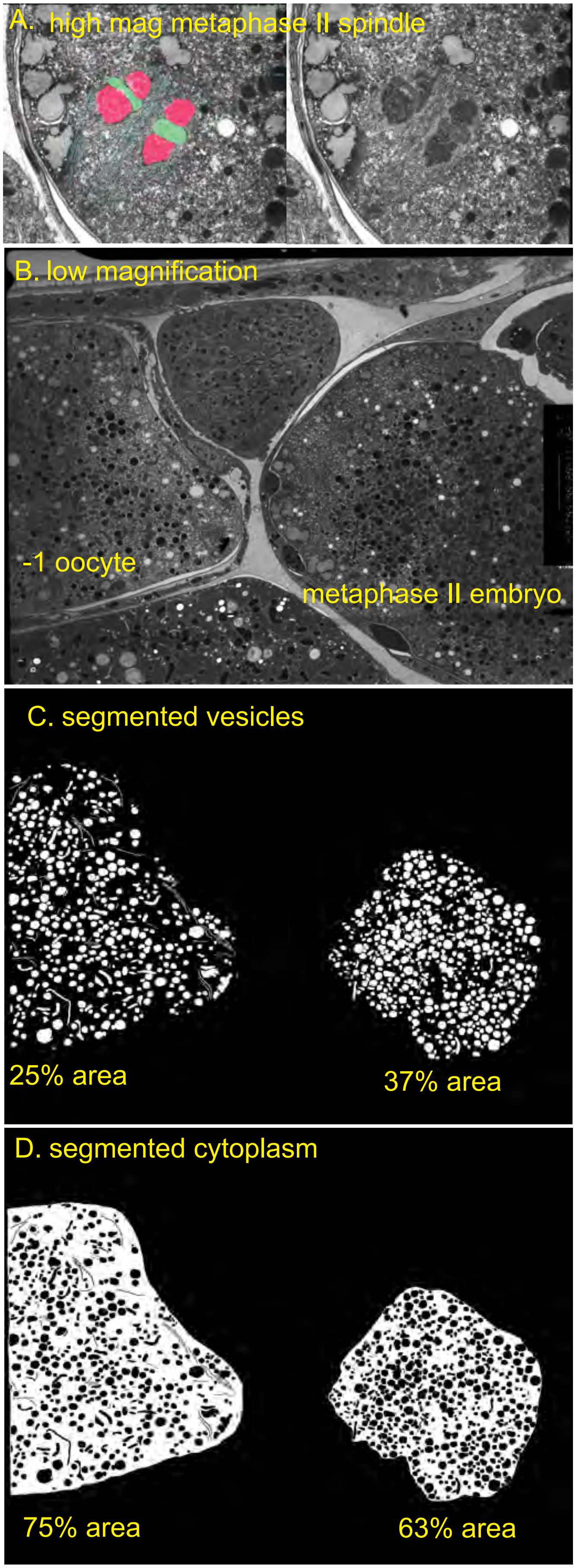

Fig. S4. Cytoplasmic volume determined from transmission electron micrograph. (A) High magnification of metaphase II spindle in the embryo on the right in B to show the quality of fixation. (B) Low magnification TEM of -1 oocyte to metaphase II embryo. (C) Manually segmented membrane vesicles of entire -1 oocyte and packed region outside the spindle in the meiotic embryo.(D) Manually segmented non-vesicle regions.

bioRxiv preprint doi: https://doi.org/10.1101/2024.04.19.590357; this version posted April 20, 2024. The copyright holder for this preprint (which was not certified by peer review) is the author/funder, who has granted bioRxiv a license to display the preprint in perpetuity. It is made available under a CC-BY 4.0 International license.

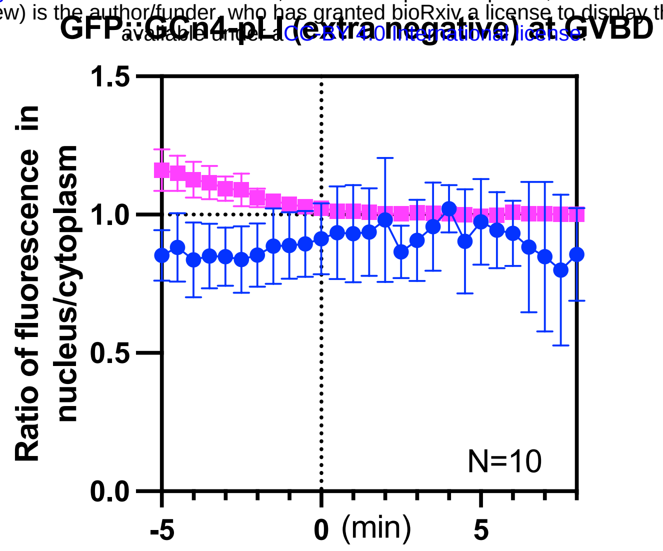

Fig S5. Movement of GFP::GCN4-pLI (extra negative) during GVBD.
Plots of fluorescence intensity ratio in nucleus to cytoplasm over time in -1 oocytes expressing mCh::histone and GFP::GCN4-pLI (extra negative) during GVBD. Y axis: mean fluorescence intensity [nucleus-background] ÷ mean fluorescence intensity [cytoplasm-background]. N: number of time lapse sequences analyzed. Mean is shown in solid magenta square [His] or solid green circle [GFP].
